# Patients’ preferences regarding physicians’ gender: a clinical center cross-sectional study

**DOI:** 10.1590/1516-3180.2021.0171.R1.08062021

**Published:** 2022-01-17

**Authors:** Carolina Matté Dagostini, Yan De Assunção Bicca, Miguel Bertelli Ramos, Sabrina Busnello, Murillo Cesar Gionedis, Natália Contini, Asdrubal Falavigna

**Affiliations:** I Undergraduate Medical Student, Department of Neurosurgery, Universidade de Caxias do Sul (UCS), Caxias do Sul (RS), Brazil.; II Undergraduate Medical Student, Department of Neurosurgery, Universidade de Caxias do Sul (UCS), Caxias do Sul (RS), Brazil.; III Undergraduate Medical Student, Department of Neurosurgery, Universidade de Caxias do Sul (UCS), Caxias do Sul (RS), Brazil.; IV Undergraduate Medical Student, Department of Neurosurgery, Universidade de Caxias do Sul (UCS), Caxias do Sul (RS), Brazil.; V Undergraduate Medical Student, Department of Neurosurgery, Universidade de Caxias do Sul (UCS), Caxias do Sul (RS), Brazil.; VI Undergraduate Medical Student, Department of Neurosurgery, Universidade de Caxias do Sul (UCS), Caxias do Sul (RS), Brazil.; VII MD, MSc, PhD. Coordinator, Health Sciences Undergraduate Program, Universidade de Caxias do Sul (UCS), Caxias do Sul (RS), Brazil.

**Keywords:** Patients, Physicians, Cross-sectional studies, Sexism, Gender inequality, Patients’ preferences, Physicians’ gender

## Abstract

**BACKGROUND:**

Even with the significant growth of female representation within medicine, inequality and prejudice against this group persist.

**OBJECTIVE:**

To analyze patients’ preferences regarding the gender of physicians in general and according to different specialties, and the possible reasons behind their choice.

**DESIGN AND SETTING:**

Cross-sectional study at the Clinical Center of the University of Caxias do Sul, Brazil.

**METHODS:**

Over a three-month period in 2020, 1,016 patients were asked to complete a paper-based 11-item questionnaire.

**RESULTS:**

The majority (81.7%; n = 830) of the patients did not have a preference regarding the gender of physicians in general. The preference rate for same-gender physicians was 14.0% (n = 142/1,016), and this preference was more common among female than among male patients (17.6% versus 7.0%; odds ratio, OR = 2.85; 95% confidence interval, CI = 1.80-4.52; P < 0.001). When asked about their preference for the gender of the specialist who they were waiting to see, the overall preference rate for a same-gender professional was 17.2% (n = 175). Preference for same-gender specialists was higher for specialties essentially based on pelvic or breast examination (i.e. gynecology, urology, proctology and mastology), compared with others (33.4% versus 9.7%; OR = 4.69; 95% CI = 3.33-6.61; P < 0.001).

**CONCLUSIONS:**

The patients’ model for choice of their physician does not seem to involve physicians’ gender in general or in the majority of medical specialties. The data presented in this study may make it easier to understand patients’ preferences and concerns.

## INTRODUCTION

Gender disparity is defined as a social phenomenon in which discrimination against others occurs due to their gender (male or female).^1^In the field of healthcare, women represent 70% of the worldwide workforce, and this percentage has increased sharply over recent years.^2^Currently, even with the significant growth of female representation in field of medicine, inequality and prejudice against this group persists.^3,4^It has been shown that female residents are notably more likely to be mistreated, both by patients and hospital staff, which may lead to higher rates of burnout syndrome and suicidal thoughts among this gender, compared with male colleagues.^5^

The idea that patients will choose their healthcare provider based on gender is an issue that has been discussed in the literature, albeit to a limited extent. Some previous studies have shown that patients seem to have a predilection in favor of male physicians for general medical care.^6–9^Among the medical specialties of obstetrics and gynecology, most patients were found to report a female preference when selecting this specialist.^10,11^In contrast, other studies within the emergency department and orthopedic specialties revealed that there was neither any patient preference for the physicians’ gender, nor any propensity towards same-gender physicians.^12,13^Some authors have argued that the reasons behind this divergence in the literature may encompass factors such as cultural and regional influences, as well as the specialty studied.^14,15^

While the representativeness of women in medicine has already been widely discussed and studied, few publications have focused on patients’ views on the topic.^3^The literature still lacks studies that have assessed patient reception in the light of the increasing numbers of women in the most varied medical specialties. Analysis on patients’ preference for male or female physicians within clinical care is an important tool to be considered in studies on patients’ perceptions, since this elucidates gender disparities regarding physicians in the field of healthcare.

## OBJECTIVE

The aim of this study was to analyze patients’ preferences regarding physicians’ gender in general and according to different medical specialties, at a single center, along with the possible reasons behind their choice.

## METHODS

### Study design and location

A cross-sectional study was conducted using a paper-based questionnaire on patients’ preference for physicians’ gender. It was carried out between October and December 2020, at the Clinical Center of UCS (Centro Clínico, Universidade de Caxias do Sul, CECLIN-UCS), a public secondary-level healthcare center for medical specialties, in Caxias do Sul, Rio Grande do Sul, Brazil. The adult medical specialties present at CECLIN-UCS at the time of the study were: urology, general surgery, nutrology, cardiology, general surgery, cardiac surgery, vascular surgery, thoracic surgery, plastic surgery, bariatric surgery, dermatology, endocrinology, gastroenterology, geriatrics, gynecology, hematology, infectiology, mastology, nephrology, neurology, orthopedics, ophthalmology, otorhinolaryngology, pneumology, proctology and rheumatology. Data collection took place through a paper-based questionnaire that was designed and distributed to patients by the researchers.

### Ethics committee

This study was previously approved by the Research Ethics Committee of the University of Caxias do Sul (CEP-UCS), under protocol number 29785920.7.0000.5341, approved on April 13, 2020. Prior to application of the questionnaires, each patient gave written informed consent to use of their information in clinical studies. The principles of the Helsinki Declaration were followed.

### Population studied

To be included, patients needed to: (1) be waiting for an appointment at CECLIN-UCS; (2) be ≥ 18 years old; and (3) agree to participate in the study by signing the free and informed consent statement. Incomplete questionnaires were excluded.

### Sample size calculation

For the purposes of sample size calculation, we considered a significance level of 5%, absolute error of 5% and population size of 50,000 people, corresponding to the average annual attendance at CECLIN-UCS. The resulting sample size was 382 individuals.

### Questionnaire on patients’ preference for physicians’ gender

This paper-based 11-item questionnaire written in Portuguese (Attachment 1) was anonymous. It was divided into three sections: (1) general information; (2) patients’ preference for physicians’ gender in general; and (3) patients’ preference for physicians’ gender according to medical specialties.

The general information section asked about the individual's age, biological sex, sexual orientation, marital status, level of education, monthly income expressed as Brazilian minimum wages per month, which was 1045.00 reais in 2020, and medical specialty within which the patient was being seen. The sections on patients’ preference for physicians’ gender in general and patients’ preference for physicians’ gender according to medical specialties included two questions each. The former asked about the individual's preference for the gender of physicians in general and the reasons for this preference. The latter also asked about preference and reasons, but specifically in relation to the specialty within which the patient was waiting for the appointment.

### Outcomes

The primary outcome consisted of the patients’ preference for the physicians’ gender in general. The secondary outcomes were: (1) the patients’ preference for the physicians’ gender according to medical specialties; (2) reasons for gender preference; and (3) comparison of gender preference between male and female patients.

### Statistical analysis

We used IBM SPSS Statistics for Windows, version 23.0, released 2015 (Armonk, New York, United States: IBM Corp.). Age presented asymmetrical distribution (P < 0.001 in the Kolmogorov-Smirnov test) and was presented both as the median ± quartile deviation and as the mean ± standard deviation and its respective 95% confidence interval (95% CI). Age means were compared using the nonparametric Mann-Whitney test. Age groups were defined in terms of quartiles. Categorical variables were presented as frequencies and percentages. Comparisons of these variables were made using the chi-square test or Fisher's exact test. The significance level was set at 0.05. The crude and adjusted odds ratios (OR and AOR) were obtained by means of binary logistic regression. The model for the preference for same-gender physicians in general considered the following variables relating to the participants: gender (male or female), age group (< 44, 44-55, 56-65 or > 65 years) and educational level (up to complete elementary school or at least incomplete high school).

## RESULTS

### Demographic data

Among the 1,041 questionnaires received, 1,016 were complete and were therefore included in the analysis. The median and mean ages of the respondents were, respectively, 55.0 ± 10.9 years and 54.3 ± 15.4 years, ranging from 18 to 93 years (P25 = 44.0; P50 = 55.0; P75 = 65.75). The majority of the patients were women (66.0%; n = 671) and self-reported that they were heterosexual (94.7%; n = 962). The most frequent marital status was “married” (47.9%; n = 487). Regarding schooling, 66.9% (n = 680) had not completed high school education. Most of the respondents (89.1%; n = 905) had an income of up to two Brazilian minimum wages per month. Table 1shows the detailed demographic data on the participants.

**Table 1:** Detailed demographic data on the participants

Variable		Frequency (%)
Sex		
	Female	671 (66.0)
	Male	345 (34.0)
Age		
	< 44 years	251 (24.7)
	44-55 years	260 (25.6)
	56-65 years	251 (24.7)
	> 65 years	254 (25.0)
Sexual orientation		
	Heterosexual	969 (95.4)
	Homosexual	22 (2.2)
	Bisexual	22 (2.2)
	Asexual	3 (0.3)
Marital status		
	Single	215 (21.2)
	Married	583 (57.4)
	Divorced or widowed	218 (21.4)
Educational level		
	No formal education	43 (4.2)
	Incomplete elementary school	363 (35.7)
	Complete elementary school	159 (15.6)
	Incomplete high school	115 (11.3)
	Complete high school	208 (20.5)
	Incomplete university education	77 (7.6)
	Complete university education	51 (5.0)
**Monthly income** a		
	< 1 minimum wage	303 (29.8)
	1-2 minimum wages	602 (59.3)
	> 2 minimum wages	111 (10.9)
**Specialty of the appointment**		
	Gynecology	172 (16.9)
	Gastroenterology	142 (14.0)
	Cardiology	105 (10.3)
	Vascular surgery	93 (9.2)
	Endocrinology	76 (7.5)
	Urology	63 (6.2)
	Mastology	53 (5.2)
	General surgery	40 (3.9)
	Proctology	35 (3.4)
	Pneumology	35 (3.4)
	Nephrology	29 (2.9)
	Bariatric surgery	27 (2.7)
	Ophthalmology	22 (2.2)
	Other^b^	124 (12.2)
	**Total**	**1,016 (100.0)**

a Income is expressed as Brazilian minimum wages per month

b Other refers to specialties with ≤ 20 responses (rheumatology, neurology, otolaryngology, nutrology, dermatology, geriatrics, hematology, cardiac surgery, plastic surgery, orthopedics, thoracic surgery, infectiology, oncology and hepatology).

### Patient preference for physician gender in general

**Figure 1:**
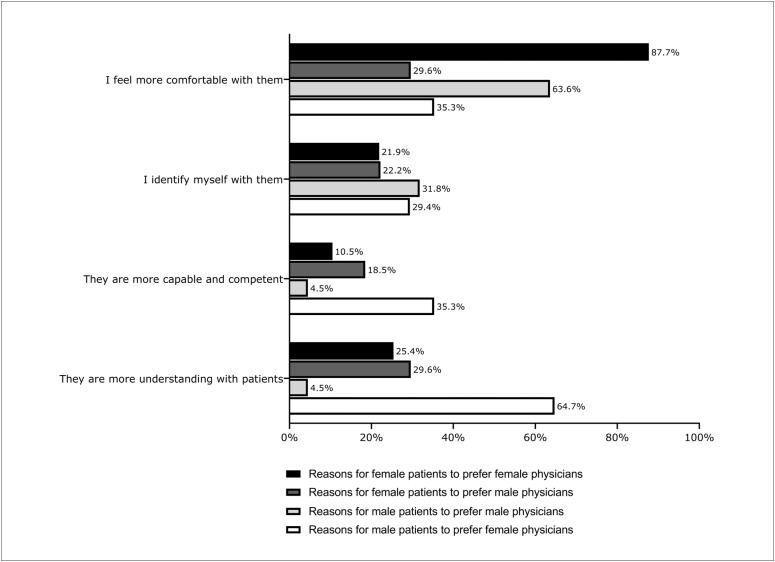
Reasons for preference for male and female physicians in general according to patients’ gender.

The majority (81.7%; n = 830) of the patients did not have a preference regarding the gender of physicians in general (Table 2). The rate of preference for same-gender physicians was 14.0% (n = 142/1,016), and this preference was more common among female patients than among male patients (17.6% versus 7.0%; AOR = 2.56; 95% CI = 1.60-4.10; P < 0.001) (Table 3). Women were more likely to prefer female physicians than were men (17.6% versus 4.9%; OR = 4.12; 95% CI = 2.43-6.97; P < 0.001). Men, in turn, were slightly more likely to prefer male physicians than were women (7.0% versus 4.0%; OR = 1.78; 95% CI = 1.01-3.14; P = 0.04). Figure 1illustrates the reasons behind the preference for male or female physicians according to patient gender. The most common reason for preferring same-gender physicians was “feeling more comfortable with them”.

**Table 2:** Patients’ preference regarding their physicians’ gender, stratified according to the gender of the patient

Patients’ gender	Patients’ preference – frequency (%)		
	Prefer male physicians	Prefer female physicians	No preference
Male	24 (7.0)	17 (4.9)	304 (88.1)
Female	27 (4.0)	118 (17.6)	526 (78.4)
Overall	51 (5.0)	135 (13.3)	830 (81.7)

**Table 3:** Preferences for same-gender physicians stratified according to sex, age group and educational level

Variable		Preference for same-gender physician			
		Frequency (%)	OR (95% CI)	AOR (95% CI)	P-value
Sex					
	Female	118/671 (17.6)	2.85 (1.80-4.52)	2.56 (1.60-4.10)	< 0.001
	Male	24/345 (7.0)	Reference category		
Age group					
	< 44 years	58/251 (23.1)	Reference category		
	44-55 years	34/260 (13.1)	0.50 (0.31-0.80)	0.47 (0.29-0.76)	0.002
	56-65 years	17/251 (6.8)	0.24 (0.14-0.43)	0.24 (0.13-0.44)	< 0.001
	> 65 years	33/254 (13.0)	0.50 (0.31-0.79)	0.48 (0.29-0.82)	0.006
Educational level					
	Up to complete elementary school	79/565 (14.0)	Reference category		0.08
	At least incomplete high school	63/451 (14.0)	1.00 (0.70-1.43)	0.70 (0.46-1.04)	
	Total	142/1,016 (14.0)	**N/A**	**N/A**	**N/A**

OR = crude odds ratio; AOR = adjusted odds ratio.

The mean age of the patients who preferred same-gender physicians was lower (49.3 years; 95% CI = 46.4-52.2) than that of those who did not have a preference (55.12 years; 95% CI = 54.1-56.1) (P < 0.001). The age group with the highest preference for same-gender physicians was the youngest group (< 44 years) (Table 3). Those who had a level of education up to complete elementary school did not have a statistically significant difference regarding preference for physicians’ gender, compared with those who had at least incomplete high school education (14.0% versus 14.0%, AOR = 0.70; 95% CI = 0.46-1.04; P = 0.08) (Table 3).

### Patients’ preference for physicians’ gender according to medical specialties

**Figure 2:**
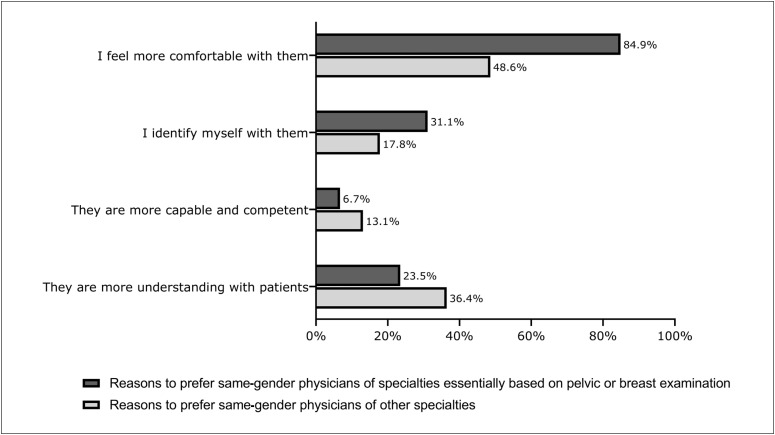
Reasons for preference of same-gender specialists for specialties that are essentially based on pelvic and breast examination, and for other specialties.

When asked about the gender of the specialist who they were waiting to see, the overall rate of preference for a same-gender professional was 17.2% (n = 175). For specialties that are essentially based on pelvic or breast examination (i.e. gynecology, urology, mastology and proctology), patients were more likely to prefer same-gender specialists, compared with other specialties (33.4% versus 9.7%; OR = 4.69; 95% CI = 3.33-6.61; P < 0.001). Among specialties with more than 20 responses, the highest preferences were observed for gynecology (41.3%; n = 71/172), urology (27.0%; n = 17/63), proctology (22.9%; n = 8/35), mastology (22.6%; n = 12/53) and general surgery (22.5%; n = 9/40) (Table 4). Figure 2illustrates the reasons behind the preference for a same-gender specialist for specialties essentially based on pelvic or breast examination and for other specialties. The most common reason was “feeling more comfortable with them”.

**Table 4:** Preference for same-gender specialists stratified according to specialty and patients’ gender

Specialty	Preference for same-gender specialists – n/total (%)		
	All patients	Female patients	Male patients
Gynecology	71/172 (41.3)	71/171 (41.5)	0/1 (0.0)
Urology	17/63 (27.0)	2/11 (18.2)	15/52 (28.8)
Proctology	8/35 (22.9)	7/18 (38.9)	1/17 (5.9)
Mastology	12/53 (22.6)	12/52 (22.2)	0/1 (0.0)
General surgery	9/40 (22.5)	7/25 (28.0)	2/15 (13.3)
Endocrinology	11/76 (14.5)	9/52 (17.3)	2/24 (8.3)
Gastroenterology	15/142 (10.6)	12/80 (15.0)	3/62 (4.8)
Nephrology	3/29 (10.3)	2/15 (13.3)	1/14 (7.1)
Ophthalmology	2/22 (9.1)	2/13 (15.4)	0/9 (0.0)
Vascular surgery	7/93 (7.5)	3/57 (5.3)	4/36 (11.1)
Bariatric surgery	2/27 (7.4)	1/24 (4.2)	1/3 (33.3)
Cardiology	5/105 (4.8)	2/53 (3.8)	3/52 (5.8)
Pneumology	1/35 (2.9)	1/22 (4.5)	0/13 (0.0)
Other^a^	12/124 (9.7)	9/78 (11.5)	3/46 (6.5)
**Total**	**175/1,016 (17.2)**	**140/671 (20.9)**	**35/345 (10.1)**

a Other refers to specialties with ≤ 20 responses (rheumatology, neurology, otolaryngology, nutrology, dermatology, geriatrics, hematology, cardiac surgery, plastic surgery, orthopedics, thoracic surgery, infectiology, oncology and hepatology).

## DISCUSSION

Our findings suggest that most of the patients did not have a preference regarding the gender of physicians in general. It was also demonstrated that preference for same-gender physicians was higher among female patients than among male patients. For specialties essentially based on pelvic or breast examination (i.e. gynecology, urology, proctology and mastology), compared with others, there was a marked preference for specialists of the same gender.

The proportion of women in the medical profession has increased over recent decades, and more markedly so over recent years.^16–18^According to data from the World Health Organization (WHO), women make up about 70% of the worldwide workforce within the field of healthcare.^2^Reports on medical demographics in Brazil in 2020 showed that women accounted for 46.6% of physicians in this country, and that in three Brazilian states (Rio de Janeiro, Pernambuco and Alagoas), their proportion already surpassed 50%.^17^Among younger physicians, female gender predominates, with 58.5% in the age group up to 29 years old, and 55.3% in the group between 30 and 34 years old.^17^Women's representation within medicine started to increase in 1970 and continued to grow until 2009, when they first surpassed men to represent the majority of medical professionals, accounting for 50.4% of all registered physicians.^18^Since then, this proportion has continued to progressively increase, reaching 57.5% in 2019.^17^

The difficulties that women encounter when entering surgical residency are commonly associated with factors such as long training, pre-existing prejudice in the surgical environment, lack of credibility in their abilities and prejudices stemming from patients and family members alike who believe in the tradition of male dominance within surgical specialties.^5,19–23^In evaluating discrimination, abuse, harassment and burnout outcomes in surgical residency programs, Hu et al.^5^found that acts of mistreatment, both from patients and hospital staff, occurred more often against women; 65.1% of female respondents reported gender discrimination and 19.9% recounted sexual harassment. That study also revealed that mistreatment was an impactful factor in the development of burnout syndrome (38.5%) and suicidal thoughts among residents (4.5%), and that women were more likely than male colleagues to report burnout symptoms (42.4% versus 35.9%; odds ratio, 1.33; 95% CI, 1.20 to 1.48).^5^

Nonetheless, a cohort study carried out by Huang et al. revealed that the two genders demonstrated similar diagnostic efficacy.^24^Thus, it appears that the gender-based inequality between physicians does not stem from differences in clinical and diagnostic skills.^25–27^Even though many of these challenges are still encountered by women when choosing a residency program, this scenario seems to be changing for the better. As Dineen et al.^13^remarked in their findings, medicine as a whole has seen a tremendous rise in female representation over the past years, albeit at a slower pace within surgical specialties.

Regarding patients’ preference for the gender of their healthcare provider, previous studies have shown that in most cases, there is no tendency towards either males or females.^6,12,28^Kerssens et al., in a study developed in the Netherlands, showed that among 961 patients questioned about their preference for physicians’ gender, there was virtually no difference with regard to the majority of healthcare professionals.^29^Likewise, our results showed that most patients did not have any preference regarding the gender of physicians in general (81.7%) (Table 2).

In contrast, Greene et al.^6^carried out a cross-sectional survey among 915 patients in the United States to investigate whether there would be any preference based on the physician's name alone. They found that the group analyzed had a predilection in favor of male names for their medical care provider, although this was not statistically significant (46.5%; P = 0.19). Moreover, Dineen et al.,^13^in another survey in the United States evaluating patients’ preferences when selecting orthopedic providers, found that 14.5% of the patients preferred a female surgeon and that, among these respondents, 89.2% of them were women. In our study, the rate of predilection for a same-gender physician in general was 14.0%, and it was 17.2% when considering preference according to the specialty within which the patient was waiting for a consultation, and this was more frequently observed among females. We also observed that the age group with the highest preference for same-gender physicians was the youngest (< 44 years). The mean age among those who had this tendency was 49.3 years, versus 55.12 years among those who did not (Table 3). The prevailing reason for preferring same-gender physicians was “feeling more comfortable with them” (Figure 1).

Despite advances in gender equality within medicine, some medical fields still have higher prevalence of men or women among their specialists.^28,30^In Brazil, women form the majority in dermatology (77.9%), pediatrics (74.4%) and endocrinology (70.6%); while in the United States female-dominated specialties comprise obstetrics and gynecology (83.4%), allergy and immunology (73.5%) and pediatrics (72.1%).^28,31–33^In Brazil, male physicians predominate in urology (97.7%), orthopedic surgery (93.5%) and neurosurgery (91.2%); while in the United States, the male-dominated specialties are orthopedic surgery (84.6%), neurosurgery (82.5%) and interventional radiology (80.8%).^28,31–33^

Previous studies revealed that within specialties based around pelvic or breast examination (such as gynecology and obstetrics, mastology, urology and proctology), preference for same-gender physicians is indeed more frequent.^9–11,27,34–37^In a systematic review of the literature, Janssen et al.^38^evaluated patients’ preference in gynecology and obstetrics and reported that 20%-25% mentioned a strong preference for a female specialist. A cross-sectional study in which the aim was to assess gender preference for care providers among urology patients revealed that 42.8% of the patients preferred a male urologist.^37^A descriptive survey evaluating male patients’ preference regarding the gender of the physician performing rectal examinations corroborated this, through showing that 51.5% of the patients indicated a preference for a male professional.^9^On the other hand, in a prospective study regarding female preferences for breast surgeon choice, 59% of the patients had no preference for the surgeon's gender.^27^

Our study pointed out that same-gender professionals attending in these fields were 4.69 times more likely to be chosen, compared with the situation in other specialties (33.4% versus 9.7%; OR = 4.69; 95% CI = 3.33-6.61; P < 0.001). Women were more than twice as inclined to choose same-gender physicians as were men (17.6% versus 7%) (Table 3). We found that 41.5 % of female patients who came for consultations within gynecology had a same-gender preference, followed by proctology with 38.9% and mastology, 22.2%. In urology, the results showed that 28.8% within the male group had a same-gender preference (Table 4).

It is worth noting that these results surprised us. We had expected to find notably higher percentages within these medical fields. The most frequent reason given for same-gender preference, in relation both to specialties that are essentially based on examination of intimate body parts and to other specialties, was “feeling more comfortable with same-gender physicians”, although this was much more prevalent for the former group than for the latter (84.9% versus 48.6%) (Figure 2). These findings are supported by existing data in the literature. Those studies revealed that female-to-female medical consultations were thought to have a more patient-centered approach, thus promoting increased involvement, while male-to-male interactions were found to be shorter and more focused on the physician's recommendations and instructions.^14,15,39^

We also found that the patients’ educational level did not seem to play any important role in gender preference. As detailed in Table 3, those who had a lower educational level (up to complete elementary school) did not show a statistically significant difference with regard to preference for physicians’ gender, compared with those who had reached a higher level (at least incomplete high school education) (14.0% versus 14.0%). Data regarding whether formal education is an influencing factor in patient predilection for their health professionals’ gender are scarce in the literature.

### Strengths and limitations

This was an original and innovative study, given that it assessed patients’ preference for physicians’ gender in a center with a wide variety of medical specialties. In addition, the number of respondents was high (n = 1,016), in comparison with similar studies.^6,8,29^Some patients found it difficult to understand the questions and respective answers if these did not represent the patients’ beliefs. In this setting, the researchers tried to clearly explain the meaning of each expression to the respondents when applying the questionnaires. Our study was also prone to selection bias. Patients who supported gender equality may have been more likely to answer the questionnaire than others who did not. Our sample also mainly consisted of patients with a monthly income lower than two minimum wages, and this may have influenced their responses and would not be generalizable to other settings. Furthermore, some specialties may have been underrepresented, with few or no respondents due to lower volume of patients per month (such as orthopedics and cardiac surgery) or because no consultations were available within our center (such as psychiatry and neurosurgery). Lastly, we emphasize that the data presented here were limited to a single center in southern Brazil and should not be fully extrapolated to other regions of this country.

## CONCLUSION

In summary, our study showed that, in general, the majority of patients (81.7%) did not have any preference for the gender of their physician. These data demonstrate that the attribute of gender is not uniformly important to all patients. Female patients seemed to prefer a same-gender physician more frequently than did their male counterparts (17.6% vs. 7%). When our patients were asked about gender preference for specialists, the rate of preference for a same-gender professional was 17.2% (n = 175). For medical specialties involving pelvic or breast examination, there was a greater tendency towards preference for same-gender professionals than was noted in relation to other fields (33.4% versus 9.7%).

The current study provides a clearer comprehension of patients’ preferences and needs. Healthcare providers may benefit from knowing their patients’ educational levels and providing counseling when planning healthcare services. Considering that in Brazil the prevalence of disadvantages and discouragement due to gender is ubiquitous among female physicians and very uncommon among male physicians in certain medical specialties, these data may help to show a change in this scenario to a more equal patient preference.

## Attachment 1 Questionnaire on patients’ preference for physicians’ gender.

Questionnaire for analysis of patients’ preference for physicians’ gender, among patients seen at the Clinical Center of the University of Caxias do Sul

1. What is your **AGE?**________________________________
2. What is your **GENDER**? Check only **ONE**option.
  ( ) Female
  ( ) Male
3. What is your **SEXUAL ORIENTATION**? Check only **ONE**option.
  ( ) Heterosexual
  ( ) Homosexual
  ( ) Bisexual
  ( ) Asexual
  ( ) Other: _______________________________________.
4. What is your **MARITAL STATUS**? Check only **ONE**option.
  ( ) Single
  ( ) Married
  ( ) Divorced
  ( ) Widowed
  ( ) Common-law marriage
  ( ) Other: _______________________________________.
5. What is your **LEVEL OF EDUCATION**? Check only **ONE**option.
  ( ) No formal education
  ( ) Incomplete elementary school
  ( ) Complete elementary school
  ( ) Incomplete high school
  ( ) Complete high school
  ( ) Incomplete university/college graduation
  ( ) Complete university/college graduation
6. What is your **MONTHLY INCOME**in minimum wages? Check only **ONE**option.
  ( ) Less than 1 minimum wage per month
  ( ) From 1 to 2 minimum wages per month
  ( ) 2 to 3 minimum wages per month
  ( ) 3 to 4 minimum wages per month
  ( ) More than 4 minimum wages per month
7. In general, which **PHYSICIAN GENDER**do you prefer? Check only **ONE**option.
  ( ) Female
  ( ) Male
  ( ) I have no preference for gender
8. Considering the answer to the previous question, **WHY**do you prefer to be seen by physicians of this gender? Tick **ALL**the options that fit your answer.
  ( ) I feel more comfortable when being seen by physicians of this gender
  ( ) I identify more with physicians of this gender
  ( ) I believe that physicians of this gender are more capable and competent
  ( ) I believe that physicians of this gender are more understanding with patients
  ( ) Other reason: ____________________________________
  ( ) I have no preference for gender
9. What **MEDICAL SPECIALTY**are you consulting with at this clinic?
  _____________________________________________
10. Considering the answer to the previous question, which gender of physician do you prefer to be seen by, in this medical specialty that you are consulting with at this clinic? Check only **ONE**option.
  ( ) Female
  ( ) Male
  ( ) I have no preference for gender
11. Considering the answer to the previous question, **WHY**do you prefer to be seen by physicians of this gender, in this medical specialty that you are consulting with at this clinic? Tick **ALL**the options that fit your answer.
  ( ) I feel more comfortable when being attended to by physicians of this gender
  ( ) I identify more with physicians of this gender
  ( ) I believe that physicians of this gender are more capable and competent
  ( ) I believe that physicians of this gender are more understanding with patients
  ( ) Other reason: ____________________________________
  ( ) I have no preference for gender
